# Bioweathering Potential of Cultivable Fungi Associated with Semi-Arid Surface Microhabitats of Mayan Buildings

**DOI:** 10.3389/fmicb.2016.00201

**Published:** 2016-02-23

**Authors:** Benjamín O. Ortega-Morales, José Narváez-Zapata, Manuela Reyes-Estebanez, Patricia Quintana, Susana del C. De la Rosa-García, Heather Bullen, Sergio Gómez-Cornelio, Manuel J. Chan-Bacab

**Affiliations:** ^1^Departamento de Microbiología Ambiental y Biotecnología, Universidad Autónoma de CampecheCampeche, Mexico; ^2^Centro de Biotecnología Genómica, Instituto Politécnico NacionalReynosa, Mexico; ^3^Departamento de Física Aplicada, CINVESTAV, Unidad MéridaMérida, Mexico; ^4^Department of Chemistry, Northern Kentucky UniversityHighland Heights, OH, USA; ^5^El Colegio de la Frontera SurCampeche, Mexico

**Keywords:** epilithic biofilms, surface microhabitat, fungi, biological weathering, oxalate, limestone, oligotrophy, semi-arid climate

## Abstract

Soil and rock surfaces support microbial communities involved in mineral weathering processes. Using selective isolation, fungi were obtained from limestone surfaces of Mayan monuments in the semi-arid climate at Yucatan, Mexico. A total of 101 isolates representing 53 different taxa were studied. Common fungi such as *Fusarium, Pestalotiopsis, Trichoderma*, and *Penicillium* were associated with surfaces and were, probably derived from airborne spores. In contrast, unusual fungi such as *Rosellinia, Annulohypoxylon*, and *Xylaria* were predominantly identified from mycelium particles of biofilm biomass. Simulating oligotrophic conditions, agar amended with CaCO_3_ was inoculated with fungi to test for carbonate activity. A substantial proportion of fungi, in particular those isolated from mycelium (59%), were capable of solubilizing calcium by means of organic acid release, notably oxalic acid as evidenced by ion chromatography. Contrary to our hypothesis, nutrient level was not a variable influencing the CaCO_3_ solubilization ability among isolates. Particularly active fungi (*Annulohypoxylon stygium, Penicillium oxalicum*, and *Rosellinia* sp.) were selected as models for bioweathering experiments with limestone-containing mesocosms to identify if other mineral phases, in addition to oxalates, were linked to bioweathering processes. Fungal biofilms were seen heavily covering the stone surface, while a biomineralized front was also observed at the stone-biofilm interface, where network of hyphae and mycogenic crystals was observed. X-ray diffraction analysis (XRD) identified calcite as the main phase, along with whewellite and wedellite. In addition, lower levels of citrate were detected by Attenuated Total Reflectance-Fourier-Transform Infrared Spectroscopy (ATR-FTIR). Overall, our results suggest that a diverse fungal community is associated with limestone surfaces insemi-arid climates. A subset of this community is geochemically active, excreting organic acids under quasi-oligotrophic conditions, suggesting that the high metabolic cost of exuding organic acids beneficial under nutrient limitation. Oxalic acid release may deteriorate or stabilize limestone surfaces, depending on microclimatic dynamics.

## Introduction

Arid lands cover a third of Earth's continental surface (Pointing and Belnap, 2012). Semi-arid lands are comparatively less well studied than deserts, although they are represented in all continents. In these environments, hydric stress limits plant life, making microbial communities the dominant biological component associated with soils or rocks in cold and hot deserts (Pointing and Belnap, 2012). Soil and rock-surface communities (termed “SRSC”) are widespread in deserts and comprise predominantly poikilohydric (tolerant to desiccation) cyanobacteria, chlorophytes, fungi, and heterotrophic bacteria, along with lichens and mosses (Pointing and Belnap, 2012). Arid lands also pose additional environmental stress to SRSC, including high temperature and high UV radiation (Gorbushina, 2007; Pointing and Belnap, 2012). Less recognized is the fact that microorganisms from arid environments must also cope with low nutrient availability, which favors the growth of dominant photoautotrophic communities (Belnap et al., 2003). SRSC form different types of assemblages as a function of prevailing climate and substratum properties. In this sense, biological soil crusts (BSCs) and epilithic biofilms are rather conspicuous on soils and rocks, respectively, while hypolithic and endolithic communities grow in more restricted areas under translucent rocks and extant cracks, particularly in hyperarid deserts (Bowker et al., 2006; Azúa-Bustos et al., 2011; Pointing and Belnap, 2012). Interestly, at the rock-surface microscale, semi-arid lands may be considered strict arid environments due to the stringent environmental conditions that lead to the proliferation of almost exclusive microbial communities, excepts when milder conditions prevail. This microhabitat is termed subaerial, at the interface between mineral surfaces and the atmosphere; microorganisms inhabiting this microhabitat have been collectively termed subaerial biofilms (Gorbushina, 2007). This study was based in the Yucatan peninsula, Mexico, a semi-arid region (Orellana et al., 2009) as it fulfills the accepted criterion of aridity index reflecting moisture deficit (Barrow, 1992). In this region, we studied biofilms formed in the Mayan archeological site of Chichen Itza (20° 40′ 56″ N–88° 34′ 05″ W), a complex of pyramids, buildings, ball courts and pillars.

Microbial communities growing associated with stone monuments are diverse and comprise phototrophs (Ortega-Morales et al., 2001; Crispim et al., 2003; Karsten et al., 2007), bacteria (chemolithotrophic and chemoorganotrophic), archaea and fungi (Kussmaul et al., 1998; Mansch and Bock, 1998; Piñar et al., 2001; Ortega-Morales et al., 2004; Sterflinger, 2010). Studies based on cultivation, microscopy, and molecular biology have shown that Mayan limestone monuments have been found to be predominantly colonized by photoautotrophic microorganisms, in particular cyanobacteria (Ortega-Morales et al., 2000, 2005, 2013; Gaylarde et al., 2007; Ramírez et al., 2010). This trend is consistent with the view that, given the carbon-limited nature (oligotrophic) of rocks and soils in arid lands, photoautotrophic organisms tend to dominate microbial communities (Belnap et al., 2003; Pointing and Belnap, 2012). A consequence of endolithic phototroph growth is that minerals are weathered both by mechanical means and by metabolite excretion (Gaylarde et al., 2012).

Assuming that the built environment is oligotrophic (Wainwright, 1993), nutritional stress has seldom been considered a factor affecting microbial weathering in lithic niches. Therefore, this study examined fungal weathering of limestone under experiments mimicking low nutrient conditions (oligotrophy) on buildings on the semi-arid climate of the Yucatan (Ortega-Morales et al., 1999). We hypothesized that naturally-occurring fungi do not use organic acids to weather limestone surface microhabitats. We focused on fungi for three fundamental reasons: (1) they have been comparatively less studied than phototrophs in Mayan monuments; (2) although not dominant in terms of biomass, they are numerically abundant reaching 10^5^ CFU per gram of stone in Mayan monuments (Ortega-Morales et al., 2000); (3) they are recognized relevant agents in Earth processes that include the alteration and weathering of rocks (Gadd, 2007).

## Materials and methods

### Site description and sampling strategy

Chichen Itza (meaning “at the mouth of the well of the Itzaes” -former native people-) is perhaps the most emblematic Mayan archeological site in Mexico. Chichen Itza was occupied by the Maya from the Classic to the Post Classic period. The Temple of Kukulkan, the Feathered Serpent God, is the most representative monument at Chichen Itza. This ninety-foot tall pyramid was built directly upon the foundations of previous temples and has been recently recognized as one of the New Seven Wonders of the World. Mayan stone buildings were built with limestone blocks of various shapes and sizes. Building walls were coated with stucco, a mixture of burnt limestone, sand, and water (Littmann, 1960). Today, most walls have lost their stucco coatings, leaving the bare stone exposed to weathering process as The Instituto Nacional de Antropología e Historia (INAH), National Anthropology and History Institute is responsible for all preservation activities in Mexico. It manages 110,000 historic buildings and monuments, along with 29,000 officially registered archeological sites, from which 25 are Nation's World Heritage Sites. INAH has offices for the registration of monuments and archeological sites, underwater archeology, cultural property restoration, museums and exhibits. One major duty is regulation of foreign archeological research permit granting and restoration projects, among others. INAH reviews domestic and foreign archeological research projects in the country (King, 2011). The regional office of INAH Yucatan granted sampling permission, and allotted a restoration professional who was in charge of accompanying us during the field work to verify that non-destructive methods were used to recover samples.

**Figure 1:**
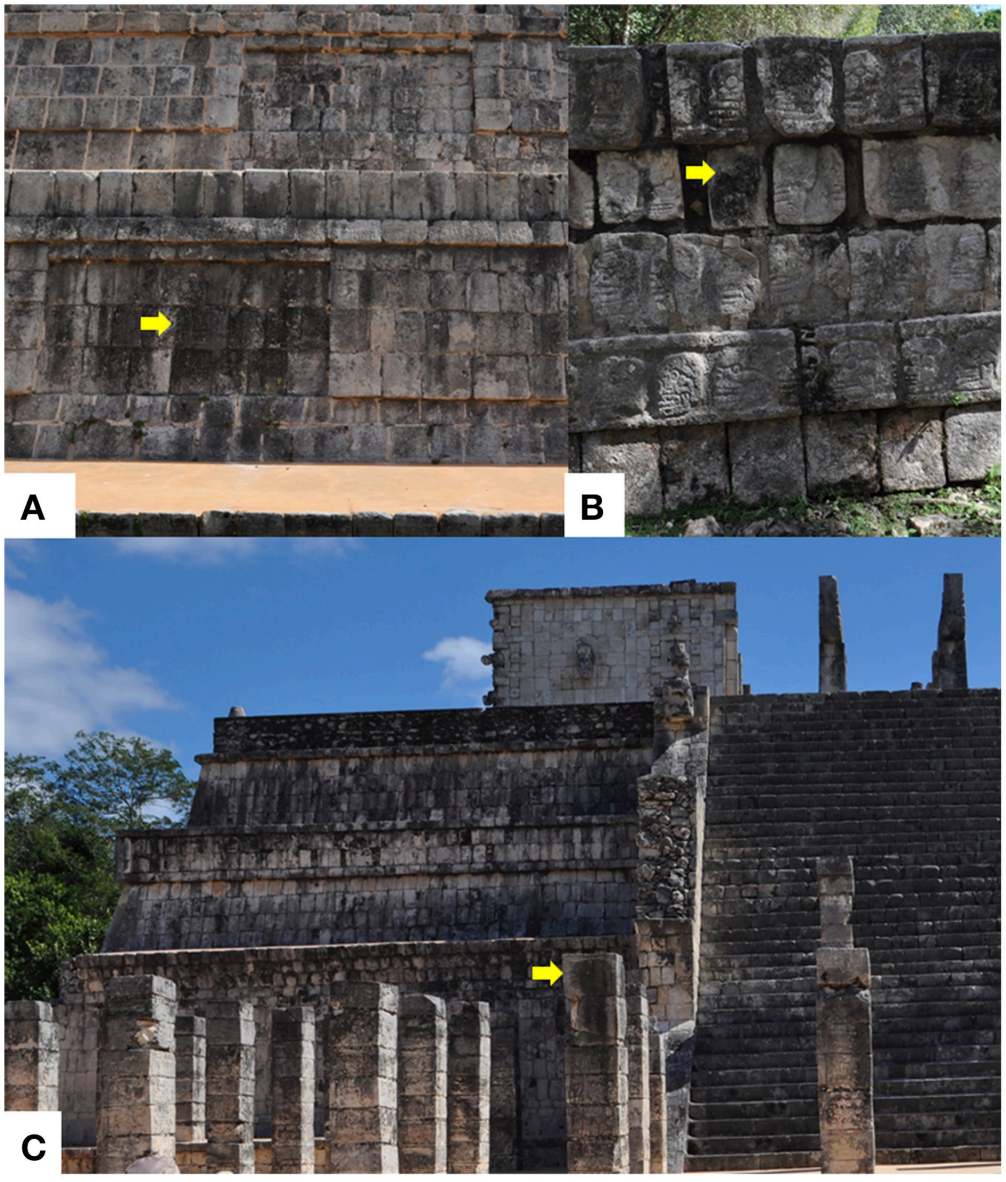
**Epilithic microbial biofilms colonizing exposed walls at Chichen Itza, Mexico: (A) Front face of Kukulkan temple; (B) Zompantli temple (Dead's House); (C) Warrior building pillars**. The microbial growth is restricted to surfaces where run-off water is available. Differential colonization here evidenced by the different degrees of blackening as indicated by the yellow arrows.

Two major types of biofilm colonize buildings at Chichen Itza, namely black biofilms (more abundant) and pink biofilms. These have been shown to be dominated by cyanobacteria (Gaylarde et al., 2007) and the chlorophyte *Trentepohlia* (Ortega-Morales et al., 2013). Extensive microbiological stainings, in particular that which darkens surfaces, is typical in historic buildings (Viles, 2002). For this reason, we restricted our sampling effort to black biofilms from several buildings. Three biofilm samples were recovered from blackened walls of temple of Kukulkán (20° 41′ 3″ N–88° 34′ 38″ W), Warriors Pillars (20° 40′ 59″ N–88° 34′ 1″ W) and the temple of Zompantli (The Dead's House) located at 20°41′ 4″ N–88° 34′ 10″ W, one from each monument (Figures 1A–C). Samples were gently scrapped off from surfaces by means of sterile scalpels, and stored at ambient temperature in the dark until laboratory analysis was possible (~12 h). Previous studies have shown that this is the best strategy for preserving microorganisms from walls in tropical or sub-tropical environments (Gómez-Cornelio et al., 2012). In this study, airborne fungi were not analyzed for comparative purposes. This Analysis could not be carried out due to limited access to the site. However, this did not alter our study's main conclusions.

### Isolation and storage of fungi

In order to isolate fungi that are predominant, by derived from mycelial fragments of biofilm biomass, or those adhering to rock particles, we proceeded to perform fungal isolations employing the method of particle filtration that includes extensive washing of particles to decrease spores (Bills et al., 2004). This method favors isolation of mycelial fragments while reducing the recovery of colonies emerging from spores (Ruibal et al., 2008). The rationale behind this choice is that by using this method we would be recovering true fungal biofilm formers and not settled fungal airborne spores. We have successfully used this technique to recover fungi from young experimental biofilms in the past (Gómez-Cornelio et al., 2012). We have termed the Diluted Suspended Biofilm method (DSB) for when spores were not intended to be eliminated by washing, whereas the Washed Biofilm Particles method (WBP) included extensive washings before fungi were allowed to grow from particles after incubation in proper media (see below). We did not attempt to describe the origin of specific isolates from particular monuments or surfaces of thus samples were pooled and split into two subsamples to isolate fungi by two different isolation strategies, as described below.

The first series of ~0.5 g subsamples were ground to particles (~200 μm) in a sterile mortar and pestle under sterile condition (laminar hood) and then added to a sterile tube. Samples were then diluted (1:10) in 0.9% saline solution. For each sample, aliquots of 100 μl were spread homogenously per quintuplicate onto 2% Malt Extract Agar (Fluka®) added/with 25 mg/l Rose bengal Sigma-Aldrich®[MEARB], 2% ME (Difco) with agar 15 g/l (Difco) [MEA], and R2A agar (Difco) [R2A-A]. All media were supplemented with chloramphenicol (100 mg/l). Plates were incubated up to 3 weeks at 27°C in the dark (Wollenzien et al., 1995; Bills and Foster, 2004). Care was taken to isolate slow-growing fungi that are commonly overgrown by fast growers. This isolation method was termed Diluted Suspension of Biofilm (DSB).

The second series of samples was first washed in saline solution (0.9%) to lower the abundance of airborne spores. Samples were ground in a mortar and subjected to three additional washings and centrifuged at 4000 rpm for 5 min. The supernatants were always discarded. The resulting particles were aseptically transferred to sterile filter paper and dried at 27°C. One hundred particles were inoculated onto MEARB, MEA, and R2A-A plates (ten particles per plate per medium) and incubated up to 3 weeks at 27°C in the dark. Plates inoculated using both methods were inspected daily up to 4 weeks. This isolation method was termed WBP. Incubation under dark conditions helps limit growth of photoautotrophic organisms (da Rocha et al., 2015).

All fungal colonies were transferred to tubes of medium MEA until obtaining pure cultures. Fungal isolates were preserved in 2% Malt Extract (ME) with glycerol 10% at −40°C and MEA slants at 4°C.

### Identification of fungi by cultivation and morphological analysis

Fungi isolates were identified according to macroscopic characteristics such as colony diameter, texture, pigmentation, margin appearance, shape, coloration, zonality, texture, density, colony elevation and production of exudates in the medium, to establish a morphotype classification, and taxon boundaries (Collado et al., 2007). Microscopic features were evaluated by observing slides prepared from secondary cultures attending to determine color, size, and morphology of the vegetative and reproductive structures. Non-sporulating fungi were sub-cultured on media such as cornmeal agar (CMA), potato dextrose agar (PDA), and oatmeal agar (OA) to promote sporulation. Non-sporulating fungi were not identified and were termed sterile mycelia (except for certain isolates that were identified by molecular methods). When identification keys were available and our expertise allowed, sporulation fungal isolates were identified to species level using the taxonomic keys of Ellis (1971, 1976), Carmichael et al. (1980), Domsch et al. (1980), Pitt (2000), Klich (2001). Unidentified sporulating fungi were termed as “unidentified fungi.”

### Molecular identification of fungi

Molecular analysis was employed to identify selected fungi. Selected isolates were grown in 100 ml of ME amended with CaCO_3_ (2 g/l) up to 10 days at room temperature, in a rotary shaker at 150 rpm. Mycelia were harvested by filtration through a filter paper and were washed with distilled water. Fresh mycelia (100 mg) were homogenized in liquid nitrogen and transferred to a 1.5 ml tube containing 600 μl of urea buffer (1.02 g/l urea, 5 M NaCl, 0.5 M EDTA, 1 M Tris HCl [pH 8.0], 0.5 g sarcosine), and vortexed briefly. The homogenate was incubated at 37°C for 30 min with RNase (10 mg/ml). After incubation, 600 μl of a phenol/chloroform (1:1) mixture was added. The sample was then vigorously shaken for 10 min, centrifuged at 5031 g for 10 min, and the supernatant transferred to a new 1.5 ml tube containing one equivalent volume of absolute ethanol and 0.1 volume of 3 M sodium acetate. The sample was mixed manually and centrifuged at 9861 g for 2 min. The pellet was washed with ethanol (70%) and then air-dried. The pellet was resuspended in 100 μl of sterile distilled water. ITS1 and ITS2 fungal universal primers were used to amplify the ITS1–5.8S–ITS2 rDNA region of the 28S rDNA (White et al., 1990). The PCR products were gel-purified with a GeneClean® II kit (Bio 101, Vista, CA, USA) and subsequently used as the template in sequencing reactions. Sequencing analysis was conducted with an ABI 377 DNA sequencer by using a BigDye® terminator cycle sequencing ready reaction kit according to the instructions of the manufacturer (Applied Biosystems, USA). The sequences were firstly aligned and compared with all the available DNA sequence databases through the Internet using the Basic Local Alignment Search Tool (BLAST; Altschul et al., 1990). The D1/D2 region of the 28S rDNA was analyzed in three fungi, isolatesTM1H05 and TM1H19 (obtained from washed sample) and *Penicillium oxalicum* Currie and Thom TM1H52 (obtained from washed sample). The first two isolates were chosen given that they were unidentified and the most active organisms in the calcium carbonate-solubilizing plate assay (see below) while *P. oxalicum* Currie and Thom TM1H52 was the most active fungus derived from unwashed sample, thus, likely derived from spores. Primers NL1/NL4 were used as previously reported (O'Donnell, 1993). PCR products were gel-purified, sequenced, and analyzed as previously described.

### Accession numbers

Sequences of isolates cluster representatives were deposited at National Center of Biotechnology Information (NCBI), under accession numbers KU307268 to KU307275 and FJ810798 to FJ810882 (http://www.ncbi.nlm.nih.gov/nuccore/), according to the protocol established by NCBI.

### Organic acid-based calcium carbonate weathering under nutrient-limiting conditions

Microbial metabolic products are responsible, at least in part, for the deterioration of carbonate stone (Scheerer et al., 2009). We tested the hypothesis that naturally occurring fungi do not exert organic acid-based weathering under low nutrient conditions (oligotrophy). For this aim we used the calcium carbonate-solubilizing plate assay. Two media of different nutrient content, MEA (copiotrophic) and R2A-A (oligotrophic) were amended with 2 g/l of calcium carbonate (CMEA and CR2A-A, respectively), phenol red was used as indicator of solubilization. Positive mineral solubilizing ability was revealed by the presence of clear (halo) areas around colonies. The production of mycogenic crystals, derived from fungal metabolism, is indicative of calcium carbonate weathering and also biomineralization (Paris et al., 1995; Sayer et al., 1997). *Aspergillus niger* van Tieghem ATCC 16888, a known organic acid producer, was used as control. Plugs of fungal growth were inoculated in CMEA and CR2A-A plates and incubated at 27°C up to 28 days. Mycogenic crystals were observed by optic microscopy (40 and 100X) at different intervals (4, 7, 14, 21, and 28 days).

### Oxalate determination in mycogenic crystals

In order to verify that organic acid-mediated weathering of carbonates occurred with the subsequent production of oxalate mycogenic crystals, total oxalates concentrations were determined according to the protocol of Tuason and Arocena (2009). This analysis was carried out only for qualitative purposes to confirm whether isolates were actually producing oxalate-based crystals. Oxalate has been successfully detected using ionic chromatography (Borrelli, 1999).

Soluble and total oxalates in mycelia and agar were homogenized in either water (for soluble oxalate) or 1.5 M HCl (for total oxalate) and then heated for 10 min in a boiling water bath. Samples treated with HCl were further diluted to 0.5 M and centrifuged (4000 rpm by 20 min), and the supernatant was filtered through a (0.20 μm) pore-size nylon filter. This solution was injected directly in a Metrohm 883 Basic IC Plus Chromatograph (Metrohm, Zwizerland) equipped with a conductimetric cell as detector. A 0.4 × 25 cm Metrosep A Supp 7 column was used with a 0.4 × 4 cm Metrosep C 4 guard column. The mobile phase was sodium carbonate (5 mmol/l) aqueous solution pumped at a flow rate of 0.800 ml/min. The regeneration liquid for conductivity suppression was an aqueous solution of sulfuric acid 100 mmol/l. Integration and calibration was performed by MagIC Net 2.3 software (Metrohm, 2010–2016).

### Experimental colonization and weathering of limestone by selected fungal isolates

Three-milliliter of mycelial suspensions TM1H05, TM1H19, TM1H26, and TM1H52 were inoculated into 250 ml Erlenmeyer flasks containing 50 ml of ME. Freshly-quarried limestone samples (coupons), ~1 cm^3^ in volume, were surface-sterilized using UV light exposure for 48 h after autoclaving at 121°C for 6 h and 105°C in oven for 24 h (Gómez-Cornelio et al., 2012). Sterile coupons were aseptically introduced into the flasks and incubated at 27°C up to 14 days at 50 rpm. The coupons were removed at two time points (4 and 14 days) and fixed in an aqueous solution of glutaraldehyde (5%) during 10 min. Coupons were air-dried overnight and placed in a desiccator until analyzed to visualize the fungal biofilm formation by scanning electron microscopy (SEM) and to characterize the production of mycogenic crystals resulting from limestone weathering activity (X-ray diffractometry). The samples for SEM analysis were fixed to polymer stubs, gold-sputtered, and examined in a JEOL JM-6360 microscope operating at 20 Kv, whereas the surface of biofilm-colonized and bulk powdered samples were scanned from 2–75° 2θ, counting for 1 s per 0.02° step on a Siemens D5000 theta/theta diffractometer using Co Kα radiation selected by a diffracted beam monochromator. The identification of crystalline phases was performed by matching of patterns in the International Centre for Diffraction Data (ICDD), Powder Diffraction File 2 (PDF2) release 2001.

Given that major mycogenic crystals made up of calcium oxalate on calcareous substrates (Burford et al., 2006) may mask other minor calcium carbonate transformation products derived from fungal metabolism, attenuated total reflectance Fourier transform infrared (ATR-FTIR) spectroscopy was employed identify microbial metabolic products on carbonate surfaces (Bullen et al., 2008). Erlenmeyer flasks containing 50 ml of ME inoculated with isolates TM1H05, TM1H19, TM1H26, and TM1H52 were incubated at 27°C in a rotary shaker at 50 rpm for 4 and 8 days. Abiotic controls were included. The spent culture broths were filtered with gauze and Nitrocellulose filter of 0.45 μm pore size to remove fungal biomass. Sterile coupons were then placed into the flasks containing the filtered medium and left under static conditions overnight to allow the limestone to react with dissolved metabolites. Coupons were then removed, dried in a desiccator and analyzed. ATR-FTIR spectroscopy measurements were made on treated samples and coupons untreated limestone (dry control) with a Nicolet Nexus 670 FTIR spectrometer equipped with a DTGS detector and a MIRacle ATR accessory with a 45° Si/ZnSe crystal (Pike Technologies, Watertown WI USA). Data collection and spectral calculations were performed using OMNIC software. Spectra were taken at 64 scans and with a resolution 4 cm^−1^.

### Statistical analysis

In order to determine the influence of nutrient levels (oligotrophic vs. copiotrophic media) in carbonate-weathering activity and subsequent mycogenic crystal formation, one-way analysis of variance (ANOVA) was conducted using the software STATISTICA 7.0 (StatSoft Inc., Tulsa, OK, USA).

## Results

### Influence of isolation strategy on fungi composition

In this study, the composition of the cultivable fungal community derived from black biofilms, typical of external limestone monuments in Yucatan Peninsula was determined. This type of microbial biofilm dominates the soiling coverage of external limestone surfaces in the Mayan region (Gaylarde et al., 2007) and is particularly conspicuous in North-oriented walls, sloped surfaces, or in vertical areas where water is available most of the time through run-off (Figures 1A–C). A total of 101 isolates representing 52 different taxa were studied (Table 1). Six out of 52 taxa were identifiable species (11%), while a large proportion of fungi remained identified only at the genus level (40%). Sterile mycelia represented roughly a quarter of total organisms detected (28%), less abundant fungi could only be ascribed to a higher taxonomic rank class Sordariomycetes (2%), and Sphaeropsidales (2%). Overall the cultivable fungal community was represented by fungi belonging to eight orders, from which Hypocreales (48%) and Xilariales (24%) accounted for over 70% of the identified isolates. These were followed by representatives of Eurotiales (10%). The remaining isolates were less well represented in the orders Botryosphaeriales, Capnoidales, Pleosporales, Sordariales, Sphaeropsidales with 3% of isolates each.

**Table 1:** **Fungal isolates from limestone biofilms from Chichen-Itza detected by means of two methods of isolation and demonstrating carbonate weathering activity**.

**Code**	**Order**	**Working taxa**	**DSB/WBP**	**Production of crystals in media plate^b^**		
			**Methods of isolation^a^**	**CMEA**	**CR2A**	**Time^c^**
TM1H01	Sordariales	*Trichocladium canadense* **S. Hughes**	DSB/WBP	++	+	14
TM1H02		Unidentified fungus 1	WBP	+	+	28
TM1H03		Unidentified fungus 2	WBP	+	+	28
TM1H04		Unidentified fungus 3	WBP	−	−	28
TM1H05	Xylariales	*Rosellinia* sp.^*^	WBP	++++	++++	4
TM1H06		Unidentified fungus 4	WBP	+++	−	21
TM1H07		Unidentified fungus 5	WBP	++	++	21
TM1H09		Sterile micelium 1	WBP	−	−	28
TM1H10	Xylariales	*Pestalotiopsis maculans* **(Corda) Nag Raj**	DSB/IWBP	+++	+++	7
TM1H11		Sterile micelium 2	WBP	+	+	28
TM1H12	Xylariales	*Hypoxylon* sp. ^*^	WBP	+++	++	7
TM1H13		Sterile micelium 3	WBP	+++	+++	14
TM1H14		Sterile micelium 4	WBP	−	−	28
TM1H15	Hypocreales	*Trichoderma* sp. 1	DSB/IWBP	−	−	28
TM1H16		Unidentified fungus 6	WBP	+++	+++	7
TM1H17		Sterile micelium 5	WBP	−	−	28
TM1H18		Sordariomycetes	WBP	−	−	28
TM1H19	Xylariales	*Annulohypoxylon stygium* ^*^ (Lév.) Y.M. Ju, J.D. Rogers and H.M.	WBP	++++	+++	4
TM1H21		Sterile micelium 6	WBP	+++	+++	14
TM1H22		Sterile micelium 7	WBP	−	−	28
TM1H23		Sterile mycelium 8	WBP	−	−	28
TM1H25		Sterile micelium 9	WBP	−	−	28
TM1H26	Xilariales	*Pestalotiopsis microspora* ^*^ (Speg.) Bat. and Peres	WBP	+++	+++	7
TM1H27		*Xylariaceae* 1	WBP	+++	+++	7
TM1H28	Hypocreales	*Trichoderma* sp. 2	WBP	−	−	28
TM1H29		Sterile micelium 10	WBP	−	−	28
TM1H32	Hypocreales	*Trichoderma* sp. 3	WBP	−	−	28
TM1H33	Xylariales	*Xylariaceae* 2	WBP	+++	+++	7
TM1H34	Xylariales	*Xylariaceae* 3	WBP	+++	+++	7
TM1H35		Sterile micelium 11	DSB	−	−	28
TM1H36	Botryosphaeriales	Lasiodiplodia theobromae^*^ (Pat.) Griffon and Maubl.	DSB	+	−	28
TM1H37		Sterile micelium12	DSB/IWBP	−	−	28
TM1H39	Hypocreales	*Fusarium* sp. 1	DSB	−	−	28
TM1H40	Hypocreales	*Fusarium* sp. 2	DSB	−	−	28
TM1H41		Sterile micelium 13	DSB	−	−	28
TM1H42	Hypocreales	*Trichoderma* sp. 4	DSB	+	−	28
TM1H43	Hypocreales	*Trichoderma* sp. 5	DSB	−	−	28
TM1H44	Hypocreales	*Fusarium* sp. 3	DSB	−	−	28
TM1H46	Sphaeropsidales	*Sphaeropsidal*	DSB	−	−	28
TM1H47	Hypocreales	*Trichoderma* sp. 6	DSB	−	−	28
TM1H48	Hypocreales	*Fusarium* sp. 4	DSB	−	−	28
TM1H49	Hypocreales	*Fusarium* sp. 5	DSB	−	−	28
TM1H50	Hypocreales	*Fusarium* sp. 6	DSB	−	−	28
TM1H51	Hypocreales	*Fusarium sp. 7*	DSB	−	−	28
TM1H52	Eurotiales	Penicillium oxalicum ^*^ Currie and Thom	DSB	++++	++++	4
TM1H53	Pleosporales	*Cochliobolus* sp.	DSB	−	−	28
TM1H54	Hypocreales	*Trichoderma* sp. 7	DSB	+	−	28
TM1H55	Capnodiales	*Cladosporium* sp.	DSB	−	−	28
TM1H57	Eurotiales	*Paecilomyces* sp. 1	DSB	++	−	28
TM1H58	Eurotiales	*Paecilomyces* sp. 2	DSB	−	−	28
TM1H60		Sterile micelium 14	WBP	+++	++	14
TM1H61		Sterile micelium 15	WBP	+++	+++	14

a *Methods: DSB (Diluted Suspension of Biofilm), WBP (Washed Biofilm Particles)*.

b *Semiquantitative abundance of crystals was recorded as (++++) very abundant, (+++) abundant, (++) common, (+) rare, and (−) absent*.

c *Time refers to the day point when activity was first detected*.

* *Molecular data*.

Remarkably, 55% of all recovered fungi (29 morphotaxa) were exclusively isolated using the WBP method. A slightly lower number of fungi were also exclusively isolated when using the DSB method 38% (20 morphotaxa), assumed to be derived mainly from spores. Little overlap occurred for both isolation methods, as only three fungi were obtained irrespective of the method employed. This indicates that the choice of isolation method played a determinant role in cultivable fungal assessment, as different subsets of the fungal community associated with limestone monuments at Chichen Itza were accessed.

### Molecular identification by RISAof selected fungi

The two most active fungi from WBP method (TMH05 and TMH19) and the most active derived from DSB (TMH52) isolation methods along with five randomly selected of fungi were analyzed by molecular methods. ITS1–5.8S–ITS2 rDNA region was amplified from each fungus analyzed. The phylogenetic analysis of this sequence allowed us to identify 10 genera of fungi including *Annulohypoxylon, Cladosporium, Cochliobolus, Fusarium, Hypoxylon, Lasiodiplodia, Penicillium, Pestalotiopsis, Rosellinia*, and *Xylaria* and 1 class Sordariomycetes. BLAST analyses of the ITS1–5.8S–ITS2 rDNA region (a single product of 450–850 bp) showed a high homology (95–100%) to previously reported sequences. Most of the isolates were grouped on defined clades. The unidentified (by morphology) isolates TMH05 and TMH19 were identified by analysis of ITS1–5.8S–ITS2 and D1/D2 ribosomal regions as *Rosellinia* sp. and *Annulohypoxylon stygium*, respectively. Both belong to the order xylariales. Both genera are reported for the first time in the rocky substrates. The identity of the remaining isolates was confirmed for genera *Pestalotiopsis* (Xylariales), *Chloliobolus* (Pleosporales), *Penicillium* (Eurotiales), which was very active in calcium carbonate solubilization and *Lasiodiplodia* (Botryosphaeriales), and allowed the identification of TMH12 as *Hypoxylon* sp. (Xylariales).

### Organic acid-based calcium carbonate weathering under nutrient-limiting conditions

**Figure 2:**
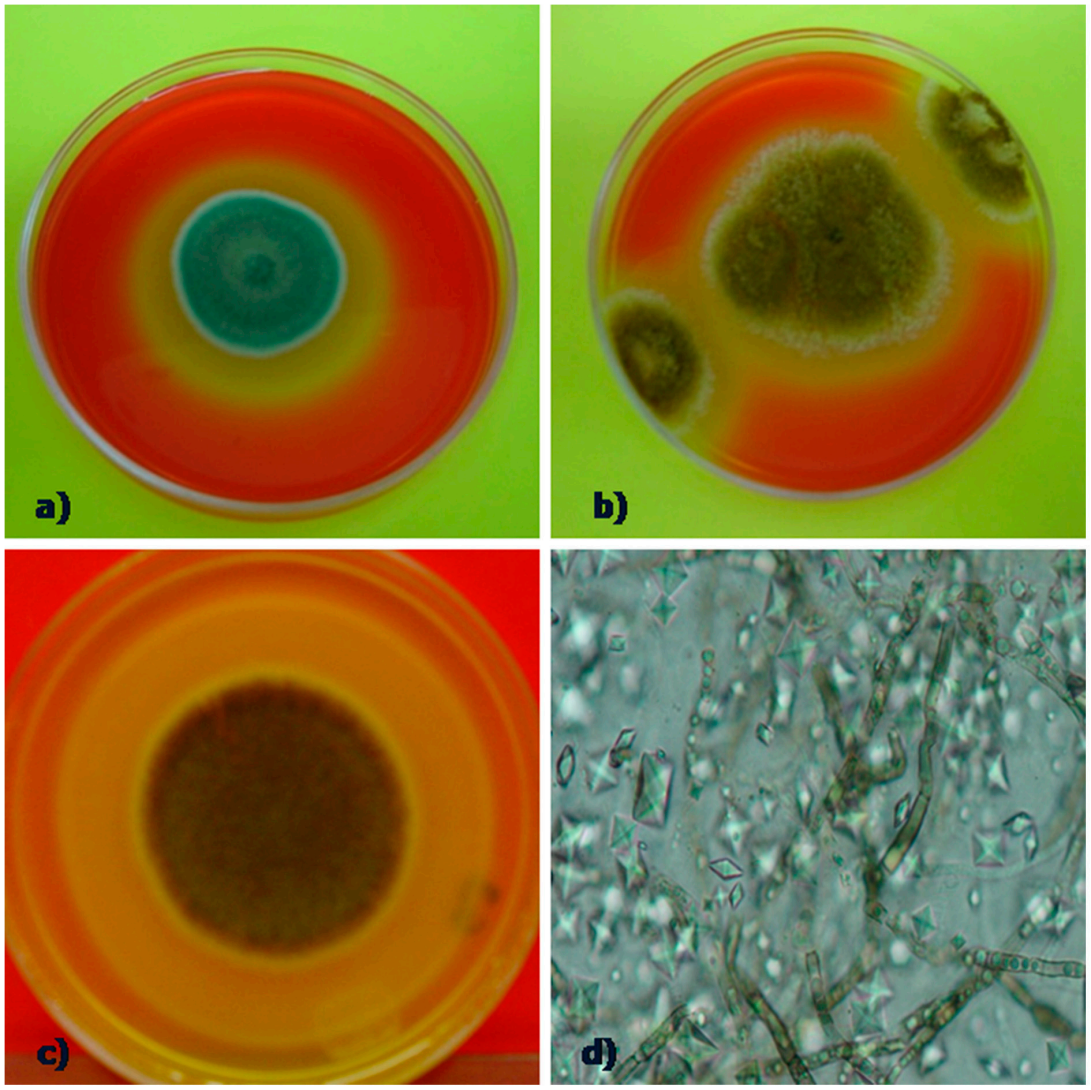
**Calcium carbonate weathering activity by selected fungi (A) *P. oxalicum*, halo indicanding carbonate solubilizing, (B) positive control (*Aspergillus niger* ATCC 16888)**. **(C)** solubilization halo forming by *Annulohypoxylon stygium* and **(D)** crystals interdispersed with fungal hyphae retrieved from the halo of solubilization zone.

The ability of fungi to solubilize calcium carbonate-amended plates was tested *in vitro*. Positive calcium carbonate-solubilizing fungal isolates was monitored by the production of a clear zone (halo) around growing colonies (Figures 2A–D). This analysis showed that the type of culture media (copiotrophic vs. oligotrophic) did not influence in general in the CaCO_3_ solubilization ability among isolates (*p* > 0.05), ruling out our proposed hypothesis. In contrast, time of incubation played a significant role (*F* = 31.6; *P* = 0.008) on the solubilization activity detected (Table 1). Certain fungi were able to solubilize carbonate early at the beginning of the incubation process (4 days) as in the case of the fungi *P. oxalicum* Currie and Thom (TM1H52) and control *Aspergillus niger* van Tieghem ATCC 16888 *A. stygium* (Lév.) Y. M. Ju, J. D. Rogers and H. M. (TM1H19) *Pestalotiopsis maculans* (Corda) Nag Raj*, P*. *microspora* TM1H26 and *Xilareaceae* TM1H33 showed wheathering activity at 7 days (Table 1). Other fungi produced a delayed response and started to solubilize the substrate by the end of the testing period (28 days). Most of the early solubilizers produced the greatest activity; the concomitant result for the solubilization of carbonates was the production of mycogenic crystals in the halo zones as evidenced by direct examination by light microscopy (Figures 2A–D). This analysis confirmed the production of morphologically different crystals interdispersed with fungal hyphae.

**Figure 3:**
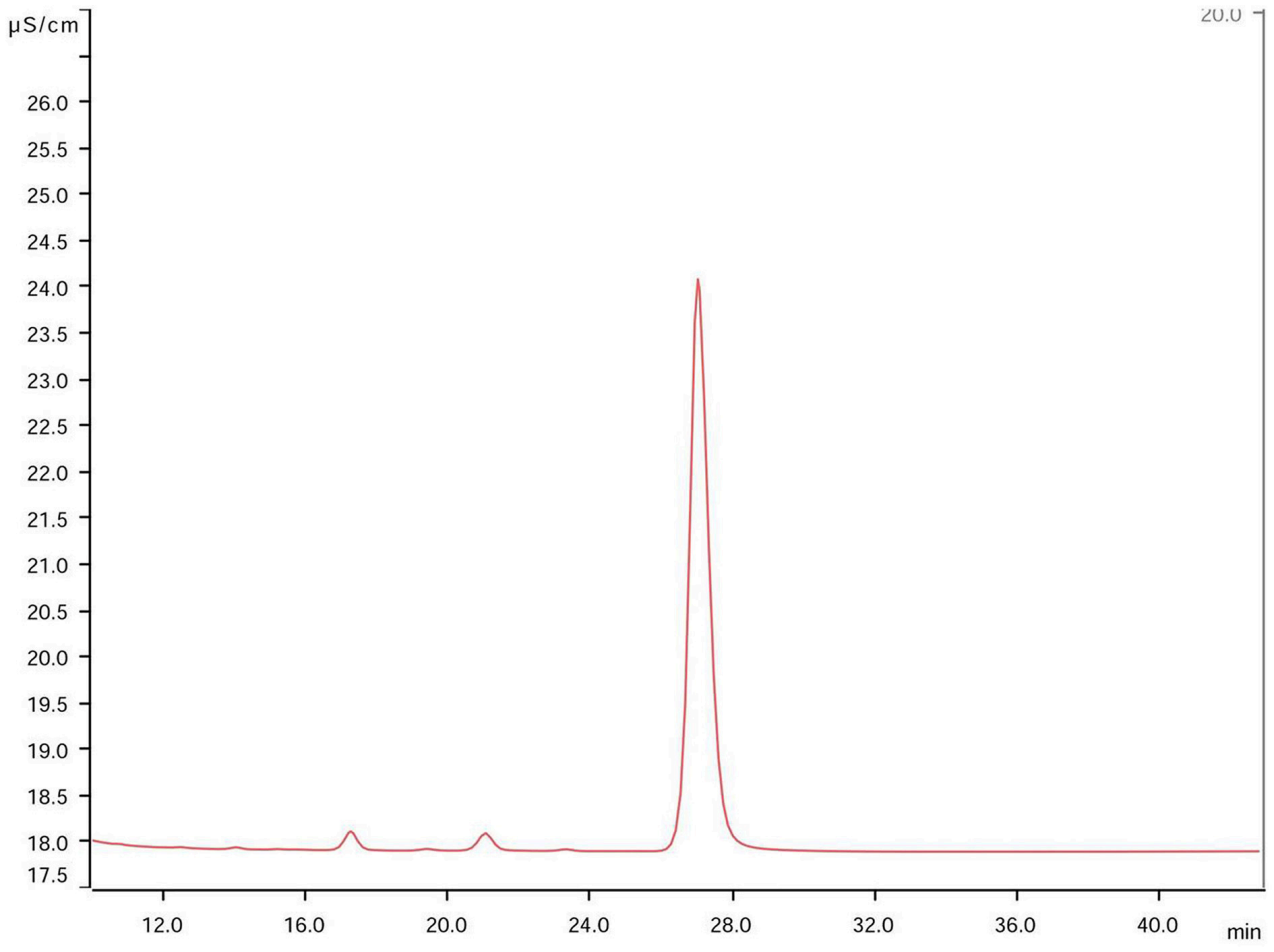
**Chromatogram of constitutive elements derived from solubilized mycocrystals produced by the fungus *Rosellinia* sp. TM1H05 on CMEA medium after 28 days**. The main peak corresponds to oxalate at 28 ± 1 min, and this peak was observed in both chromatograms of pure oxalic acid and calcium oxalate standard solutions. No other organic acid was detected.

On the other hand, the ionic chromatographic analysis of crystals extracted from the agar and mycelia revealed that oxalates made up most of their mass as no other organic acid was detected (Figure 3). Remarkably, there was a clear trend among the solubilizing capability of isolates, as most of the active fungi were those recovered by means of the WBP method (59%), while only 26% of fungi isolated using the DSB method expressed calcium carbonate-weathering activity. In addition, in most cases, this activity reduced when detected.

### Fungal transformation of limestone coupons

**Figure 4:**
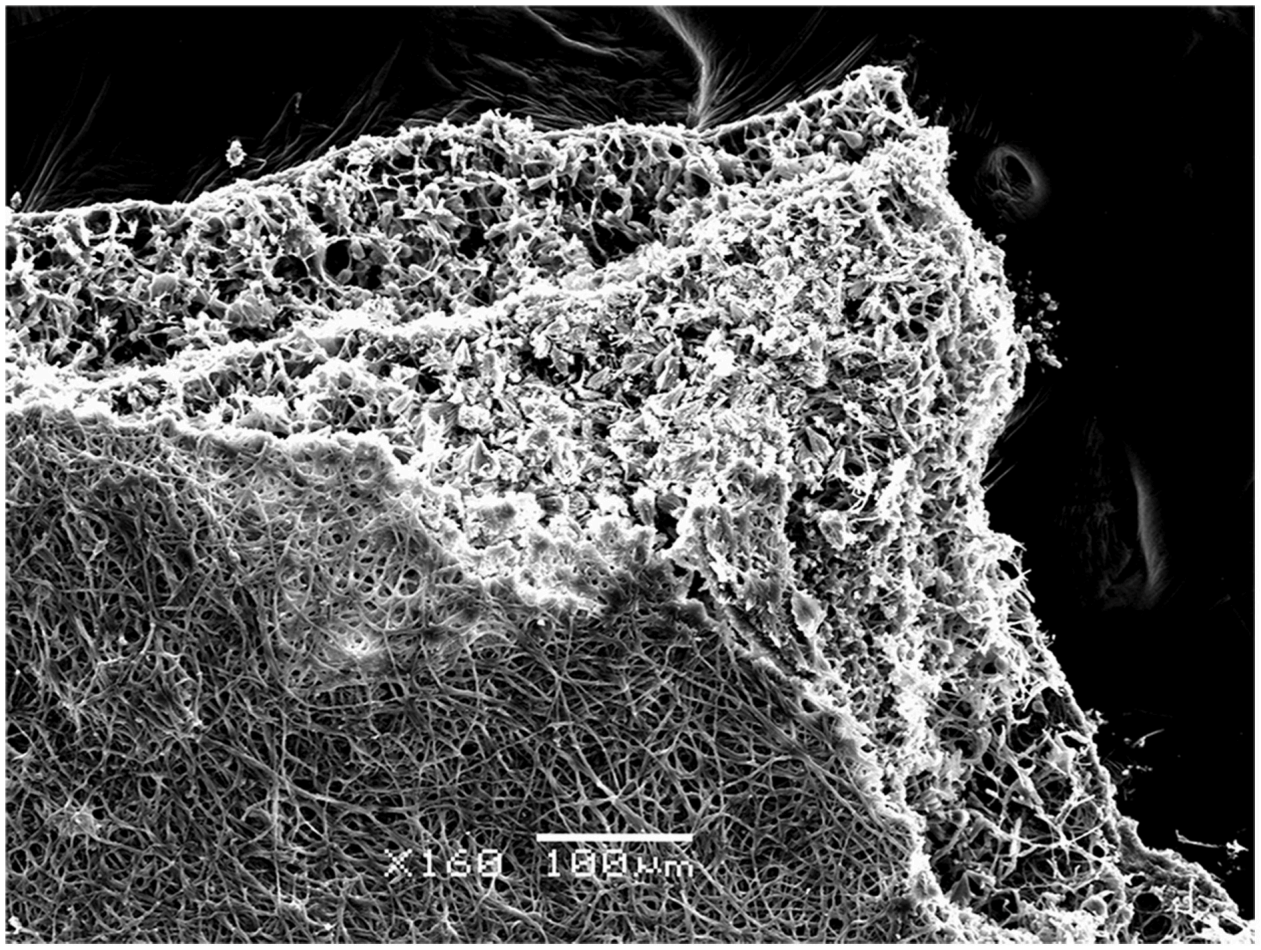
**Fungal colonization *in vitro* on limestone coupons by *Annulohypoxylon stygium* as shown by Scanning Electron Microscopy (SEM)**. Biomineralized hyphae in the bottom part of the biofilm is seen. Bar = 100 μm.

**Figure 5:**
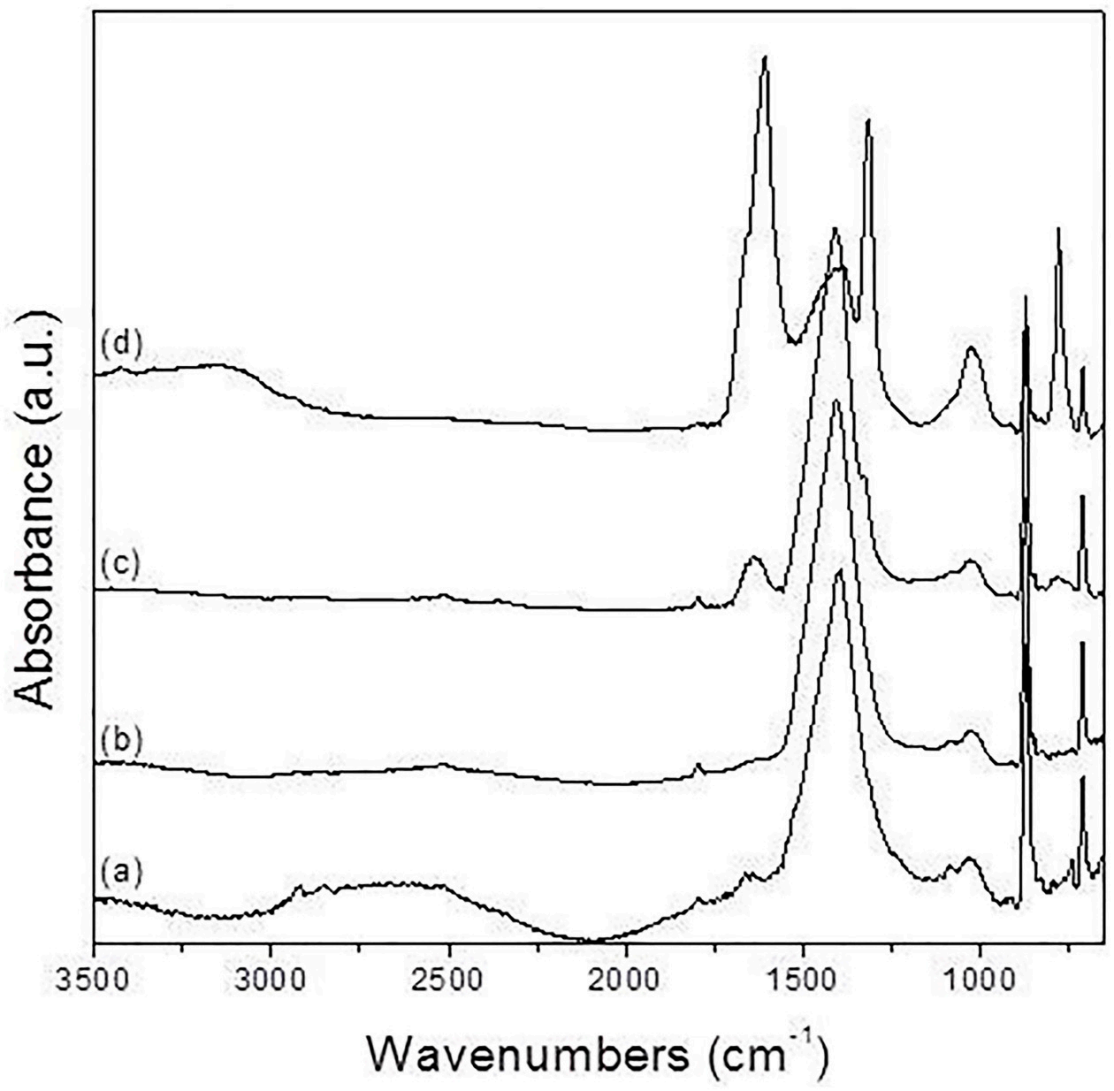
**Normalized ATR-FTIR spectra of calcite samples: (A) dry control, (B) wet control, (C) exposed to *Rosellinia* sp.TM1H05, and (D) exposed to *Aspergillus niger* ATCC 16888**.

Further testing of bioweathering activity in microcosm with *Rosellinia* sp. TM1H05, *A. stygium* TM1H19, *P. microspora* TM1H26, and *P. oxalicum* TM1H52 revealed that these fungi formed biofilms on limestone surface. Mycogenic crystals were seen adhering to the outer surfaces of fungal hyphae as in *A. stygium* (Figure 4). Crystals examined by SEM revealed that the prevalent crystals were tetragonal di-pyramids. Power X-ray diffraction of limestone coupons showed that calcite was transformed into calcium oxalates to different extents (Table 2). In addition, these mineralogical analyses confirmed that oxalates included the two polymorphs (calcium oxalate monohydrate and dehydrate) from which calcium oxalate monohydrate dominated (CaC_2_O_4_.H_2_O; whewellite). As an additional analytical tool, ATR-FTIR was used to evaluate other metabolic activity products that may occur at lower levels. Figure 5 showed the ATR-FTIR analysis of limestone coupons untreated (dry control) (1A) and were control (1B) and the coupons treated inoculated with *Rosellinia* sp. (TM1H05) (1C) and *A. niger* ATCC 16888(1D). The *A. niger* ATCC 16888 shows spectral features that were consistent with the presence of citric acid on the limestone surface. This was evident by strong bands developing at 1590 and 772 cm^−1^. *Annulohypoylon stygium* (TM1H19) shows similar bands to the *A. niger*, as evident by bands recorded at 1592 and 772 cm^−1^ and the shoulder at 1352 cm^−1^. However, these bands are much weaker in intensity, which suggests that citric acid, may develop at a lower concentration, with low surface coverage. It should be noted that the ATR-FTIR spectra for both the wet and dry controls (Figures 5A,B) were similar to each other and indicate the culture medium did not influence the spectral analysis of metabolic products.

**Table 2:** **Cultivation of selected fungi with limestone coupons and mineralogical analysis of mycogenic crystals**.

**Isolate**	**Identity of fungi**	XRD profiles^**^
TM1H05	Xylariaceae (*Rosellinia* sp.)^*^	Calcite (12%) Wheddellite (88%)
TM1H19	Sterile micelium (*Annulohypoxylon stygium*)^*^	Calcite (42%) Wheddellite (58%)
TM1H26	Pestalotiopsis microspora^*^	Calcite (12%) Whewellite (7%) Wheddellite (81%)
TM1H52	Penicillium oxalicum^*^	Calcite (5%) Whewellite (88%) Wheddellite (7%)

* *Molecular data. ARISA bands*.

** *Mineralogical composition by XRD profiles*.

## Discussion

The microbial ecosystems in semi-arid lands have received scant attention as most research has been conducted on hyper arid and arid regions (deserts). Microbial communities dominate the landscape in arid lands but studies have focused on photoautotrophic populations, the dominant component of these communities (Belnap et al., 2003; Bowker et al., 2006; Azúa-Bustos et al., 2011; Pointing and Belnap, 2012). In this regard, fungi remain understudied despite the key roles that this microbial group plays in weathering rocks, formation of soil, and cycling of nutrient (Gadd, 2007). This study contributes to advance our understanding of the diversity of fungal communities in epilithic microhabitats exposed to semi-arid climate and their potential impact on limestone weathering under nutrient-limiting conditions that mimic the rock environment.

The taxa that could be identified by morphological criteria and molecular data were predominantly representatives of the *Hypocreales* (48% of isolates) followed by *Xylariales* (24%). Fungi of the orders *Botryosphaeriales, Capnoidales, Pleosporales, Sordariales, Sphaeropsidales* were less well represented. It is difficult to determine if this observation is in agreement with a general trend for epilithic fungal communities under semi-arid conditions, as very few studies exist to contrast our findings. In addition, there is methodological complication to establish robust comparisons as the isolation procedures, culture media, and conditions of incubation vary between studies and these have been shown to influence microbial isolate recovery (da Rocha et al., 2015). However, it is possible to compare our results with those a previous study using the same isolation method (Gómez-Cornelio et al., 2012). In this study, we found a different composition of fungal community, as the dominant orders ware *Pleosporales* (40%), *Capnoidales* (24%) e *Hypocreales* with 19% of isolates Unpublished results confirm the dominance of *Pleosporales* e *Hypocreales* and the presence of *Xylariales* in lithic environments in Yucatan. In line with this trend, a review study of fungi associated with BSC, which are similar to epilithic biofilms*, Pleosporales* and *Hypocreales* dominated the fungal communities of four distinct deserts (Bates et al., 2010). Interestingly this review highlights the absence of *Xylariales* in BSC and this observation is meaningful considering that this study Bates et al. (2010) reported both cultivation-dependent and molecular data. These comparisons suggest that fungal communities of epilithic surfaces exposed to the semi-arid climate of northern Yucatan are similar to other fungal communities in arid lands, but may harbor specific organisms such the *Xylariales* that have been not reported to be dominant in rock-surfaces (Gorbushina, 2007). Assuming that cultivation conditions did not greatly influence the composition of retrieved fungi of previous studies (Bates et al., 2010; Grishkan et al., 2015), at the genus level, substantial difference could be observed between taxa in the present study and those form from arid environments. In this investigation, only *Fusarium* and *Trichoderma* were well represented while these genera along with *Cladosporium, Penicillium*, and *Alternaria* also occurred (Bates et al., 2010; Gómez-Cornelio et al., 2012; Grishkan et al., 2015). It is important to recognize that our finding do not necessarily reflect the diversity of the total cultivable fungal community as a larger range of media, and that cultivation conditions other than those employed in this work are likely needed to capture the greater “biodiversity from arid” microbial communities (da Rocha et al., 2015) and cultivation-independent studies are thus needed to complement fungal diversity assessments (Bates et al., 2010).

Notably, an important number of detected unidentified (by morphological criteria) fungi could be resolved by the molecular approach employed in this study, and this revealed the presence of novel organisms for rock environments. *Annuhypoxylon, Xylaria*, and *Rosellinia*, all representatives of the *Xylariales* are new reports for rocky habitats and arid lands. These findings expand our view of the diversity of fungal communities in the semi-arid regions where unusual taxa such as *Elasticomyces, Hyalodendron, Monodyctis, Papulospora, Septoria, Minimedusa*, and *Gliomastix* were reported (Gómez-Cornelio et al., 2012), highlighting the validity of cultivation-based approaches to study microbial diversity (da Rocha et al., 2015). In addition, cultivation studies are needed to study physiological attributes such as the potential impact of fungal metabolism on limestone. For the purpose of our study, we felt it appropriate to focus on isolation methods that allowed us to distinguish to a reasonable level of confidence between fungi assumed to occur as spores from true biofilm formers derived mainly from myceliar growth. With this in mind, we used an isolation strategy proposed by Ruibal et al. (2008) and previously used in a previous work (Gómez-Cornelio et al., 2012). Our results however, suggested that the isolation method was determinant, as the recovery of fungi from samples previously washed presented a different composition. Interestingly, when using this method, most of the sterile mycelia, unidentified fungi (*Annuhypoxylon* and *Rosellinia*) along with the genera *Pestalotiopsis, Trichocladium*, and *Lasidiplodia* were identified (Table 1). The exhaustive washing procedure allowed that spores of deposited airborne fungi were greatly removed but that probably do not represent the true biofilm fungal dwellers on limestone. This probably explains the absence in the culture plates of fast-growing fungi likely originating from spore germination, including common fungal genera such as *Fusarium, Trichoderma, Cladosporium, Penicilium*, and *Paecilomyces*, which are typically reported in other studies (Takahashi, 1997; Simonovicová et al., 2004).

Lithic substrata have been considered oligotrophic environments, with low levels of organic matter available for microbial metabolism. This view might not be totally accurate as seasonal peaks of organic input may be expected from aerial deposition and phototrophic production (cellular exudates and dead biomass). Our findings of the carbonate weathering activity of selected fungal isolates under oligotrophic and copiotophic culturing conditions showed that this activity varied among isolates, but it was not influenced by the culture conditions but was significantly influenced by time, indicating that the transformation of potential of the fungal community is taxa-dependent. This carbonate weathering activity is linked with the excretion of acidic metabolites as evidenced by XRD and ATR-FTIR. Fungal strains from monuments excreted predominantly gluconic, oxalic, citric, and fumaric acids (Braams, 1992), but did study did not report if fungi were derived from biofilms biomass or spore material. Sterflinger (2000) reports that all taxonomic groups of fungi are able to produce oxalic acid to a greater or lesser extent, although not all species or strains are able to do so (Palmer and Hirsch, 1991). Reaction of products of oxalic acid seen as the prevalence of a certain shape of crystals the tested fungi are consistent with the reported morphology of calcium oxalates (Burford et al., 2006). SEM images showed also that a biomineralization front occurred from bottom of the fungal biofilm to the exterior layers of hyphae in contact with culture medium containing the organic acids. Our FTIR analysis however, showed that citric acid was also present but at lower levels.

Two important implications emerge from these findings. First, although the production of oxalates has been shown to occur in stone environment linked to the presence of fungi and lichens, it is currently debatable if this represents only a deterioration risk. Oxalates may also protect the stone by forming a protective layer and passivating the stone. Oxalates are in fact less soluble than carbonates. The Yucatan Peninsula along with the rest of the world is experiencing rapid climatic change. Climate change is likely to have a major impact on built cultural heritage not only through abiotic exacerbation of stone deterioration, but also by increasing influencing microbial metabolic activity (Bonazza et al., 2009). Second, A non-recognized role of photoautotrophic growth on monuments is that through organic acid-production may not only contribute to bioweathering or biostabilization of limestone (Scheerer et al., 2009; Sterflinger, 2010) but also affect trophic webs in epilithic biofilms as oxalic acid and the resulting oxalates could be potential source of carbon for specialized microflora.

## Conclusion

Our findings show that at the microscale the rock-surfaces exposed to semi-arid areas harbor fungi typical of extreme arid environments from which novel organisms, such as fungal representatives of the *Xylariales*, remain to be discovered and deserve future attention. In this regard, although there is further need to carry out cultivation-independent studies to fully assess the extent of fungal diversity in this environment, our cultivation-dependent approach proved its validity. These approaches showed that biofilm associated fungi are comprised of biofilm formers and associated mycoflora probably derived from spores, suggesting that fast-growing fungi are probably overrepresented in previous studies. Furthermore, the significant organic acid-based weathering potential of biofilm-derived fungi that contrast with that displayed by associated transient fungal communities point toward the need to assess experimental biodeterioration studies using representative organisms under conditions mimicking to the greatest possible extent the real world conditions. The metabolic versatility of epilithic fungi to release organic acids under varying nutrient levels is consistent with our view that the lithic microenvironment harbors adapted in this specialized habitat.

## Author contributions

BO designed the work, contributed materials, did field work, performed the carbonate weathering activity of isolates, analyzed the data, and wrote the paper. JN did the molecular identification of isolates, contributed materials, analyzed the data, and wrote the paper. MR did the morphological identification of isolates, analyzed the data, and wrote the paper. PQ did the XRD and SEM analysis of phases and biofilms and analyzed the data. SD did field work, performed the carbonate weathering activity of isolates, and analyzed the data. HB performed the FTIR characterization and analyzed the data. SG did field work, performed the carbonate weathering activity of isolates and analyzed the data. MC contributed materials and performed oxalate determinations by IC.

### Conflict of interest statement

The authors declare that the research was conducted in the absence of any commercial or financial relationships that could be construed as a potential conflict of interest.
